# Total body irradiation with volumetric modulated arc therapy: Dosimetric data and first clinical experience

**DOI:** 10.1186/s13014-016-0625-7

**Published:** 2016-03-22

**Authors:** Andreas Springer, Josef Hammer, Erwin Winkler, Christine Track, Roswitha Huppert, Alexandra Böhm, Hedwig Kasparu, Ansgar Weltermann, Gregor Aschauer, Andreas L. Petzer, Ernst Putz, Alexander Altenburger, Rainer Gruber, Karin Moser, Karin Wiesauer, Hans Geinitz

**Affiliations:** Department of Radiation Oncology, Krankenhaus der Barmherzigen Schwestern Linz, Seilerstätte 4, 4010 Linz, Austria; Internal Department I - Hematology with Stem Cell Transplantation, Hemostaseology and Medical Oncology, Krankenhaus der Elisabethinen Linz, Linz, Austria; Division of Medical Oncology, University of Washington, Fred Hutchinson Cancer Research Center, Seattle, WA USA; Internal Medicine I - Medical Oncology, Hematology and Gastroenterology, Krankenhaus der Barmherzigen Schwestern Linz, Linz, Austria; Medical Faculty, Johannes Kepler University, Linz, Austria

**Keywords:** Total body irradiation (TBI), Total marrow irradiation (TMI), Volumetric modulated arc therapy (VMAT), Leukaemia, Organs at risk (OAR), Dose sparing, Dose homogenising

## Abstract

**Background:**

To implement total body irradiation (TBI) using volumetric modulated arc therapy (VMAT). We applied the Varian RapidArc™ software to calculate and optimize the dose distribution. Emphasis was placed on applying a homogenous dose to the PTV and on reducing the dose to the lungs.

**Methods:**

From July 2013 to July 2014 seven patients with leukaemia were planned and treated with a VMAT-based TBI-technique with photon energy of 6 MV. The overall planning target volume (PTV), comprising the whole body, had to be split into 8 segments with a subsequent multi-isocentric planning. In a first step a dose optimization of each single segment was performed. In a second step all these elements were calculated in one overall dose-plan, considering particular constraints and weighting factors, to achieve the final total body dose distribution. The quality assurance comprised the verification of the irradiation plans via ArcCheck™ (Sun Nuclear), followed by in vivo dosimetry via dosimeters (MOSFETs) on the patient.

**Results:**

The time requirements for treatment planning were high: contouring took 5–6 h, optimization and dose calculation 25–30 h and quality assurance 6–8 h. The couch-time per fraction was 2 h on day one, decreasing to around 1.5 h for the following fractions, including patient information, time for arc positioning, patient positioning verification, mounting of the MOSFETs and irradiation. The mean lung dose was decreased to at least 80 % of the planned total body dose and in the central parts to 50 %. In two cases we additionally pursued a dose reduction of 30 to 50 % in a pre-irradiated brain and in renal insufficiency. All high dose areas were outside the lungs and other OARs. The planned dose was in line with the measured dose via MOSFETs: in the axilla the mean difference between calculated and measured dose was 3.6 % (range 1.1–6.8 %), and for the wrist/hip-inguinal region it was 4.3 % (range 1.1–8.1 %).

**Conclusion:**

TBI with VMAT provides the benefit of satisfactory dose distribution within the PTV, while selectively reducing the dose to the lungs and, if necessary, in other organs. Planning time, however, is extensive.

## Introduction

Total body and total marrow irradiation (TBI, TMI) are well-established parts of several conditioning regimens required prior to bone marrow (BMT) or allogeneic stem cell transplantation (HSCT). In addition to killing malignant cells TBI/TMI suppresses the immune system and thus helps to prevent transplant rejection.

In comparison to one-dose applications hyper-fractionated treatments result in lower side effects [1, 2]. Various dose- and fractionation-schemes were used in the past [3–5]. In general total doses from 12 to 15 Gy in 6 to 11 fractions were reported (e.g. [6–9]).

Different methods are in use, such as irradiation from the right and left side, while the patient is sitting in a specially adapted chair [10, 11]; other institutes carry out irradiation in the supine and prone position with the patient lying on a fixed couch on the floor or on a moving couch with the translation of the patient through the open beam [12]. Several publications report on TBI and TMI calculations with emphasis on dose distribution [13, 14]. An excellent review on the design and development of dosimetric issues from 1980 to 2014 was published recently [15]. The authors included a few papers on organ complications and immune deficiency after TBI or TMI and/or stem cell transplantation.

The translational method was improved by Quast et al. [16, 17]. Unfortunately our treatment rooms are too small to fit the moving couch equipment. Jahnke et al. [18] use an arc approach with a fixed field size. In the above mentioned TBI techniques homogeneity may possibly be jeopardized by varying body diameters. In particular when using lateral fields the dose distribution in the lungs may be quite irregular and might differ vastly from the dose that is planned to be absorbed in that organ.

Lately two high precision techniques were introduced: Helical tomotherapy was implemented for TBI and TMI some years ago [19–22] as an approach to reduce the dose to critical organs, especially the lung. Furthermore feasibility studies were carried out for planning TMI with VMAT combined with ventro-dorsally opposed fixed beams for the legs [23], an idea which may be transferred to TBI as well.

Already in 2010 the bone-marrow- and stem-cell-transplantation-centre (SCTC) of the Elisabethinen Hospital in Linz, Austria, approached the management of our hospital to have TBI performed at our radiation oncology department. The years before patients with leukaemia had to be sent to the University Clinics in Vienna or Innsbruck, Austria, for TBI. Ambulance transport and hospitalization for some days in a foreign environment has many disadvantages for these immuno-suppressed patients: the travel included the risk of infections and implied a physical and mental burden for these critically ill patients. To change this uncomfortable situation for the benefit of the patients was the major factor to implement TBI at our institution.

After various site visits of TBI Centers in Austria and abroad two factors were decisive to use VMAT (RapidArc™): on the one hand our treatment rooms are too small to use a translational couch and on the other hand the dosimetric aspects concerning the lung seemed to us unsatisfactory both for this couch method and for the bi-lateral treatment with a patient sitting on a chair. This is why we implemented a VMAT-only-approach for standard TBI patients. During a period of several months we performed calculations on dummy CT image sets via VMAT/RapidArc™ and in July 2012 we started TBI in our first leukaemia patient using a slightly different treatment concept. In July 2013 we started to perform TBIs according to a prospective treatment plan in line with the referring hospital. An average of 5 to 7 patients per year was announced by the Elisabethinen Hospital, Linz. Until February 1st 2016 twenty patients were treated with this method.

Our approach for TBI is to use pure VMAT via multiple overlapping arcs, a method which may be of benefit for several radiation oncology centres with similar architectural circumstances. The benefit for the patients may be on the one hand no transport to centres 200 km and further away, and on the other hand a high precision 3D-treatment with the advantage – in comparison to conventional methods - of applying a homogenous total body dose and to spare the lung and, if necessary, also other organs.

Herein we report on set-up and dosimetry details as well as on early clinical data of our first seven patients treated with this VMAT-TBI.

## Materials and methods

Between July 2013 and June 2014, seven patients, three with acute lymphatic leukaemia (ALL), two with acute myeloid leukaemia (AML) and two presenting with T-cell lymphoma, underwent allogeneic stem cell transplantation following TBI based myeloablative conditioning at the Elisabethinen Hospital, Linz, Austria. Patients with ALL were treated according to the “GMALL protocol” [24]. Patients with AML received induction chemotherapy with Ara-C and an anthracyclin. The mean patient-age at TBI was 30.6 years (20–52 years). Three patients were referred in first CR (complete remission), 2 in second CR, one with partial remission and one patient suffered from relapsed T-cell lymphoma.

### Contouring

The planning target volume (PTV) was contoured using the outer body contour, excluding the lung (except a small margin of lung tissue adjacent to the ribs and spine to ensure full dose coverage of the ribs). Both lungs were contoured using the pulmonary windows. The right and left lung were contoured separately, but they were considered as one structure for lung dosimetry analysis. Small vessels extending beyond the hilar regions were included. To steer the optimizer for lung sparing small helping structures inside the lung were used. However, for dose statistics and DVH, the anatomical lungs are the relevant structures. Other organs at risk (OAR) were not routinely contoured and involved in dose optimization, except for two cases: one patient had received whole brain irradiation with 24 Gy 3.3 months before TBI; therefore brain sparing was intended. In another patient the mean dose to both kidneys was restricted due to pre-existing renal insufficiency. Additional helping structures within the overlapping regions were contoured and used for steering the optimizer leading to an improved dose distribution in those areas.

### Positioning and immobilization

**Fig. 1:**
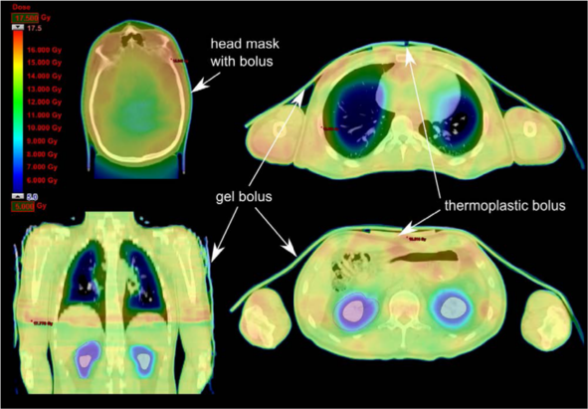
Different CT slices of the cranial part of patient H.L. The *arrows* indicate the positions of the thermoplastic and gel boluses. The colour wash presentation of the dose distribution shows the dose reductions of up to 50 % in the lung and the kidneys. In patient H.L. the kidneys were spared due to pre-TBI renal insufficiency

For each patient two computed tomography (CT) image sets (SOMATOM Sensation 16™, Siemens, Erlangen, Germany) were performed, with a slice thickness of 10 mm in the first 3 patients and of 5 mm in the following patients. Since the CT scan length of the available CT is only 160 mm, it was necessary to split the image sets into a cranial and a caudal body section to accomplish treatment of the whole body length. The patient was positioned on a vacuum mattress (VacFix™, PAR Scientific A/S, Odense, Denmark) placed into a custom made wooden box, in order to guarantee a stable mattress from the start of the planning procedure until the last treatment fraction. For the head-, neck- and shoulder-regions thermoplastic masks are used. Additional thermoplastic bolus material was placed on the head mask, the sternum, the shinbones and knees to ensure a sufficient dose to superficial bones (assigned in Fig. 1). Further, the patient was covered with elastic gel bolus mattings to achieve an adequate skin dose.

### Treatment planning and irradiation

The primarily planned dose to the overall PTV was 13.2 Gy, administered in eight fractions, which are 1.65 Gy per fraction and 3.3 Gy per day. This dose and fractionation regimen was well established at the Fred Hutchinson Cancer Research Center and Seattle Cancer Care Alliance (Seattle. WA, USA) and is a standard conditioning therapy in almost all allogeneic transplantation centres worldwide, including the Medical University Vienna (personal communications). The dose of 13.2 Gy TBI is used for transplants from an unrelated donor, in case of related donors the planned total dose is reduced to 12 Gy.

The interval between two fractions per day was a minimum of 6 h. Irradiation was performed at photon energy of 6 MV. For the lungs a mean dose of 10 Gy or lower had to be achieved. Therefore we used additional helping structures inside the lungs with low constraint values to steer the optimizer for lung sparing.

The TBI treatment plans were generated using the RapidArc™ software, provided within the Eclipse™ treatment planning system, version 10.0 (Varian Medical Systems, Palo Alto, CA) on a cluster of six T5400 workstation personal computers with 8-way 2.5 GHz Intel Pentium III processor and 24 GB of RAM. The progressive resolution optimization algorithm, version 10.0.28 (PRO, Varian Medical Systems, Palo Alto, CA), was used to optimize all RapidArc™ plans. This software version allows the simultaneous optimization of a maximum of 10 arcs in one calculation process. The final dose calculation was performed with the anisotropic analytical algorithm (AAA), version 10.0.28., using a grid size of 0.25 cm.

**Fig. 2:**
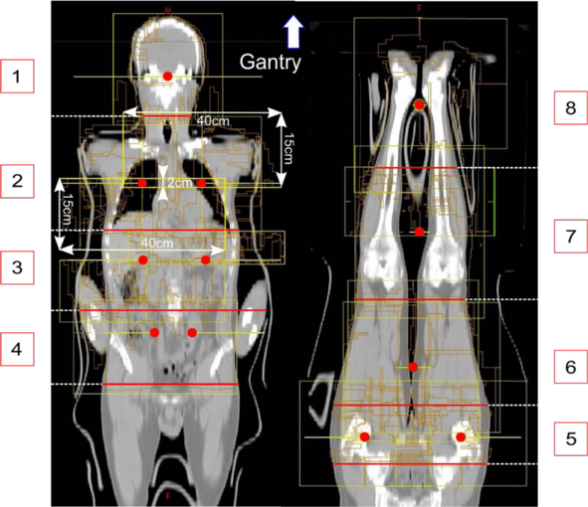
Field (*yellow rectangulars*) and iso-centre (*red dots*) arrangement: for the treatment planning the CT image set is divided into 8 segments (Seg1–8, indicated by the *red lines* and the numbers), with one iso-centre in segments 1, 6, 7 and 8, and with two coplanar iso-centres in segments 2 to 5. In segment 2, the field dimensions for 90° collimator rotation are indicated

The splitting of the planning CT images into a cranial and a caudal part necessitated a dosimetric alignment of these two body parts. The overall PTV had to be split into 8 segments (Seg_1 to 8_) with a subsequent multi-isocentric planning. The number of iso-centres was 9 to 15, dependent on the body mass index of the patient. Figure 2 shows a patient with 12 iso-centres.

**Fig. 3:**
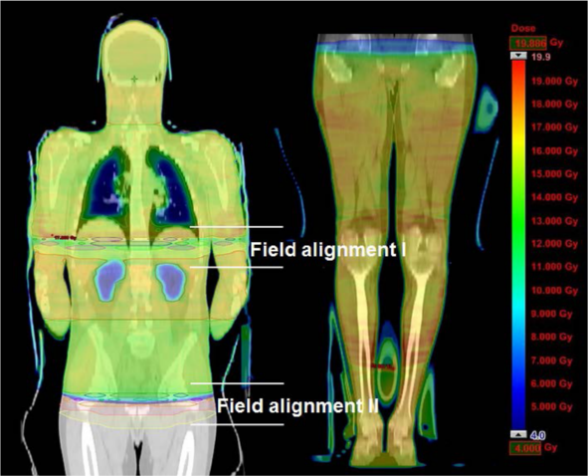
Dose distribution of the whole body (patient H.L.) in colour wash: overview of the 2 field alignment areas. Due to irregular dose distributions in these overlapping zones an extra calculation process was necessary to achieve homogenous doses and to avoid over- and under-dosages

Because of the above mentioned limitation of optimizing a maximum of 10 arcs or a total sum of 3600° within one single plan and the splitting into two CT-image groups, the treatment planning required the calculation of two field alignments, one in the lower mediastinum and the other in the lower pelvis (Fig. 3). For the proximal four segments from head to upper pelvis the patient was treated in the head first supine position. Then the patient was rotated 180° to irradiate the patient in the feet first supine position from feet to alignment zone II in the pelvis, according to segments 5 to 8. In a first step the optimization of each single segment was carried out separately. All arcs had an overlap of at least 2 cm into the neighbouring segment to avoid hot and cold spots at the junction areas. Segments with one iso-centre (Seg1,6,7,8) were planned with two full arcs (179–181°), whereas segments planned with two coplanar iso-centres (e.g. Seg2–5 in Fig. 2) were calculated with two half arcs, at each iso-centre. In general, the combination of collimator rotations of 0° and 90° were used for all arcs. The maximum field size was limited to 30 × 40 cm. In a second step all these segments (Seg1–8) had to be calculated in one overall plan.

Due to irregular dose distributions in these overlapping zones an extra calculation process was necessary to homogenize the dose using the “Convert Isodose Level to Structure”-mode of the Eclipse™ treatment planning system. These structures were used in the subsequent VMAT optimization for increasing or decreasing the dose in areas with insufficient or excessive dose, respectively. The evaluation of the final treatment plan was carried out by creating a plan sum of the cranial and the caudal CT images. Remaining over- or under dosages in the junction area between the cranial and caudal part were removed by drawing helping structures, which were used for de- or increasing the dose in these areas, respectively, in another optimization step. The final plan was accepted when no dose “islands” with doses smaller than 95 % of the prescribed dose were present inside the PTV, with the lowest dose maximum achievable.

The quality assurance comprises the verification of the dose distribution of each iso-centre via ArcCheck™ (Sun Nuclear Corporation, Melbourne, FL, USA), requiring the calculation and export of the verification plans and the measurements themselves. During treatment in vivo dosimetry is performed via an appropriate positioning of metal-oxide-semiconductor field-effect transistors (MOSFETs) on the patient. Two MOSFETs are placed in the axilla and two additional ones between wrists and hips or inguinal region to avoid any air gap between the MOSFETs and the skin. Subsequently the measured dose values are compared with the planned doses.

Before irradiation the position of the patient was verified on the basis of bony alignment for each iso-centre separately using the Varian On-Board Imager™ (OBI) kilovoltage imaging system. In most cases the position of the patient was accurate (±2.5 mm), with the exception of the arm’s position, which had to be corrected 3 times.

### Haematological cell transplantation

Three to 5 days after TBI allogeneic stem cell transplantation was performed. The patients were hospitalized at the sterile ward of the stem cell transplantation unit for 5 to 6 weeks. Patients were discharged following hematologic engraftment and in the absence of severe acute GvHD (graft vs. host disease).

## Results

The time requirement for treatment planning was high: contouring took 5–6 h, dose calculation and optimizing amounted to 25–30 h and quality assurance took 6–8 h. With regard to contouring, the manual separation of the outer contour of the patient’s body (the PTV), from the other parts like the couch, the bolus or the head mask on each of the up to 250 slices of the CT image for the cranial as well as caudal part was most time consuming. Quality assurance comprised several tasks, namely the calculation and export of the verification plans for the ArcCheck™ measurements at each iso-centre, the measurements themselves, and the MOSFET measurements during irradiation plus subsequent comparison with the planned doses. The couch-time per fraction was 2 h on day one, including step-by-step information to the patient regarding the current process, time of arc positioning, patient positioning verification with OBI, the mounting of the MOSFETs and irradiation of all segments, decreasing to around 1.5 h for the following fractions. The net irradiation time per full arc was 1 min. Two arcs were performed in each iso-centre. In the thoracic segments half arcs with a treatment time of 0.5 min each were used.

**Fig. 4:**
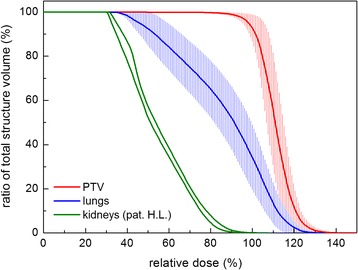
Dose volume histograms of the primary target volume (*red*) and lung (*blue*) for all patients. The *solid lines* represent the mean values; the *error bars* the standard deviation. *Green lines*: kidneys of patient H.L. (dose reduction)

Four patients received a total dose of 13.2 Gy (8 fractions twice daily). One received only 9.9 Gy (6 fractions in 3 days) due to chemotherapy toxicities. Two patients were treated to 8 Gy in 4 fractions, due to their general condition. In the patient presenting with renal insufficiency the mean dose to both kidneys could be reduced to 7 to 8 Gy (Figs. 1 and 4). In the patient with a previous brain irradiation the dose to the central brain area was reduced to 6 Gy, whereas the mean dose was restricted to 11.7 Gy.

Table 1 presents the mean dose to the PTV and the D95% of the PTV, in addition the mean doses and the D90% of both lungs, whereby D95% and D90% represent the dose coverage of the 95 % volume of the PTV and of the 90 % volume of the lung in Gy, respectively. In none of the patients the mean lung dose was above 10.7 Gy.

**Table 1:** Mean doses (in the cranial half of the patients) of PTV and OARs in Gy and dose coverage of the 95 % volume of the PTV (D95%) and of the 90 % volume of the lung (D90%) in Gy

Pat.	PTV	PTV	Lung right		Lung left	
	D_mean_	D_95%_	D_mean_	D_90%_	D_mean_	D_90%_
	Gy	Gy	Gy	Gy	Gy	Gy
D.D.	14.7	13.1	10.7	8.3	10.6	7.8
D.F.	13.9	12	10.5	5.6	10.4	5.7
E.F.^a^	11.4	10.3	9.6	7.3	9.9	7.5
H.L.	14.1	11.1	10.6	5.9	10.6	5.6
H.S.	14.5	12.8	10.5	5.8	10.7	6
E.D.^b^	9.1	8.3	6.8	5.0	6.9	4.8
L.W.^b^	9.2	8.4	7.3	4.5	7.2	4.7

^a^In patient E.F. the planned target dose was 9.9 Gy due to chemotherapy toxicities

^b^Patient E.D./L.W. was treated with 8 Gy only due to his general condition and performance status

The mean volumes in the whole body exceeding 110, 120 and 130 % of the prescribed PTV dose were 62.7 % (range 42–81.7 %), 10.8 % (range 3.5–19.7 %) and 1.6 % (range 0.13–4 %) of the PTV, respectively. All areas that received more than 120 % of the prescribed dose were outside the lungs. Small areas outside the OARs with a maximum dose of 130 % were accepted, whereas a mean of 1.6 % of the PTV received more than 130 % of the prescribed dose for all patients. The 1.6 % volume comprises all these small areas (see Table 2).

**Table 2:** Percentage of the PTV receiving 90 %, 95 %, 110 %, 120 % and 130 % of the prescribed dose, respectively

Pat.	90 %	95 %	110 %	120 %	130 %
D.D.	99.7	98.5	64.0	10.7	0.13
D.F.	98.6	97.0	42.0	3.5	0.4
E.F.	99.9	99.9	78	15.4	2.1
H.L.	99.5	98.2	49.3	5.2	3.7
H.S.	98.9	98.6	48.9	6.1	0.5
E.D.	99.0	98.5	81.7	19.7	4.0
L.W.	99.9	99.6	75.1	14.9	0.2
Mean	99.4	98.6	62.7	10.8	1.6

The planned dose was in line with the measured dose via MOSFETs: in the axilla the mean difference between calculated and measured dose was 3.6 % (range 1.1–6.8 %), and for the wrist/hip-inguinal region it was 4.3 % (range 1.1–8.1 %). In Table 2 the percentages of the PTV receiving 90, 95, 110, 120 and 130 % of the dose are listed; e.g. 98.6 % (range 97.0–99.9 %) of the PTV received 95 % of the planned dose.

### Side effects and effectiveness

The mean follow-up after TBI of all patients is 8.0 months (2.3–15.0 months). During radiotherapy all patients suffered from slight to moderate fatigue, and all presented with a pre-existing light anaemia. No other side effects during treatment were observed. Immediately after TBI all patients suffered from G3 mucositis and received high dose analgesic therapy; one patient presented with bladder inflammation for a week. No modest or severe lung reaction was observed during follow-up. One patient, who was treated during relapse without obtaining a remission after chemotherapy, died of refractory disease 2.3 months after TBI. One additional patient with ALL relapsed 8.8 months after treatment (mediastinum, neck nodes) and died after 13.4 months. The other 5 patients live disease free and without severe toxicities.

### Improvements in treatment planning

The following improvements in the planning procedures were carried out while gathering experience with VMAT-TBI: As VMAT is a high-precision irradiation technique, the slice thickness of the CT images was reduced from 10 to 5 mm to increase resolution of the dose distribution, for example in the lungs, where structure sizes (margins) as small as 10 mm were used in the dose optimization process.

The field arrangement in the thoracic region (lung) acquired special attention due to the intended lung sparing. Dividing the large 30 × 40 cm fields into two separate fields with a size of 15 × 40 cm was found to be optimum for lung sparing. The smaller dimension of the field size (15 cm) was located in the direction of the motion of the MLCs. This is indicated in Fig. 2 for a collimator rotation of 90°, where the field was separated into a cranial and a caudal part with 15 × 40 cm field size each and an overlap of 2 cm. The overlap region of 2 cm was used to guarantee a homogeneous dose in the abutting region. With this geometry, the MLCs could shield the lungs in an ideal way; additionally the smaller field sizes allowed for a better dose modulation taking advantage of the full MLC motion (e.g., over travel restriction with larger fields). When central parts of the lung received 50 % of the described dose, dose sparing was found to be optimum, however, this is only possible when using small fields.

To obtain an adequate skin dose a total-body gel bolus with a thickness of 5 mm was used from the third patient on, analogue to the use of a bolus in standard irradiation techniques.

## Discussion

VMAT generates adequate dose distributions even for complexly shaped PTVs. The challenge to use VMAT for TBI is the extremely large extension of the PTV requiring multiple overlapping arc treatments. After the initial introduction and learning phase we are at present treating about one patient per month with this technique. Admittedly, the time requirements for VMAT-TBI are high. Even with up-to-date work stations pure calculation time for plan optimization and dose calculation is very long. We hope that in the near future, the time for calculation and optimization in VMAT-TBI can be decreased due to the availability of faster computers. With regard to treatment time, 1.5 h per fraction (2 h for the first fraction) in VMAT-TBI are not far away from the treatment time using a translational couch, which is in the range of 1 to 2 h, depending on additional electron fields used to boost the ribs [25].

On the other hand VMAT-TBI results in state-of-the-art 3D-CT-based treatment planning with the option of selectively lowering or increasing the dose to particular regions of the body. Except for the lungs this might be relevant in pre-irradiated regions, in pre-existing severe organ insufficiency or –with respect to increasing the dose- in regions with a high burden of tumour. VMAT-TBI may help to reduce pneumonitis-related morbidity and mortality, but this has to be evaluated in a larger set of patients. VMAT or tomotherapy seem to be currently the best high precision techniques for 3D-TBI. VMAT/RapidArc™ and even helical tomotherapy are not able to cover the whole body without repositioning the patient, thus exact dose planning and dose delivery in these overlapping regions remains a challenging issue.

A further advantage of VMAT-TBI is that no additional devices to translate the patient through the beam are necessary. Patient positioning and beam delivery are carried out with the same system thus eliminating potential sources of error. VMAT-TBI can be delivered in any treatment room that is large enough to fit a linear accelerator.

The VMAT method described in this paper was introduced in our department in July 2012 treating a TMI case on an individual basis. TBI concept was developed in close cooperation with the local transplantation team. From June 2013 until July 2014 we applied it for TBI in 7 leukaemia patients. All of them presented with a low rate of acute side effects. For long term efficacy and late effects further follow-up is necessary.

Whether a dose reduction in the brain, the kidneys or in other organs is a remarkable improvement with regard to (late) side effects and whether these dose reductions do not lead to an increase of (extra-medullary) recurrence rates, are open questions. However, Kim et al. [26] could recently demonstrate in 101 patients receiving tomotherapy for TMI that doses lower than 10 Gy in organs at risk did not increase the rate of extra-medullary recurrences. Thus selectively reducing the dose in critical organs might be an option to reduce side effects without compromising cure rates. Patients with malignant myeloma might benefit most from VMAT, because the target volume - in comparison to TBI - may be limited to the bone marrow and the internal organs might be spared from the high dose region as much as possible [19–24, 26–29].

## Conclusion

TBI with VMAT up to 13.2 Gy in fractions of 1.65 Gy is feasible and associated with a so far low rate of early toxicity. The reason to use VMAT was twofold: The treatment rooms are too small to install a translational couch. Moreover RapidArc™ guarantees a homogenous dose to the total body and allows 3D conformal high precision RT and a selective reduction of the dose to the lungs to mean doses of 10 Gy or below. However, additional resources for treatment planning with regard to personnel and time are high. The implementation of TBI in the region is a great advantage and an additional benefit for the community. It prevents these critically ill patients from long distance to other centres and therefore prevents them from the risk of infections as well as from additional physical and mental distress.

### Ethics, consent and permissions

All patients signed, at hospital admission, consent for the use of their data for retrospective and scientific investigation.

### Ethics approval

The paper has been performed in accordance with the Declaration of Helsinki and has been approved by the local ethics committee: Ethik-Kommission des Krankenhauses der Barmherzigen Schwestern Linz Betriebsgesellschaft m.b.H.
